# Identification and Expression of the Multidrug and Toxic Compound Extrusion (MATE) Gene Family in *Capsicum annuum* and *Solanum tuberosum*

**DOI:** 10.3390/plants9111448

**Published:** 2020-10-27

**Authors:** Qinfang Chen, Linna Wang, Di Liu, Sirui Ma, Yangshuo Dai, Xue Zhang, Yuxin Wang, Tian Hu, Ming Xiao, Ying Zhou, Hua Qi, Shi Xiao, Lujun Yu

**Affiliations:** State Key Laboratory of Biocontrol, Guangdong Key Laboratory of Plant Resources, School of Life Sciences, Sun Yat-sen University, Guangzhou 510275, China; chenqf3@mail.sysu.edu.cn (Q.C.); wangln45@mail2.sysu.edu.cn (L.W.); liud47@mail2.sysu.edu.cn (D.L.); masr3@mail2.sysu.edu.cn (S.M.); daiysh6@mail.sysu.edu.cn (Y.D.); cherxuer@163.com (X.Z.); wangyx256@mail2.sysu.edu.cn (Y.W.); tianhu0401@163.com (T.H.); xiaoming3@mail.sysu.edu.cn (M.X.); zhouying25@mail.sysu.edu.cn (Y.Z.); qihua@mail2.sysu.edu.cn (H.Q.); xiaoshi3@mail.sysu.edu.cn (S.X.)

**Keywords:** MATE, Expression profile, Solanaceae, *Capsicum annuum*

## Abstract

Multidrug and Toxic Compound Extrusion (MATE) proteins are essential transporters that extrude metabolites and participate in plant development and the detoxification of toxins. Little is known about the *MATE* gene family in the Solanaceae, which includes species that produce a broad range of specialized metabolites. Here, we identified and analyzed the complement of *MATE* genes in pepper (*Capsicum annuum*) and potato (*Solanum tuberosum*). We classified all *MATE* genes into five groups based on their phylogenetic relationships and their gene and protein structures. Moreover, we discovered that tandem duplication contributed significantly to the expansion of the pepper *MATE* family, while both tandem and segmental duplications contributed to the expansion of the potato *MATE* family, indicating that *MATEs* took distinct evolutionary paths in these two Solanaceous species. Analysis of ω values showed that all potato and pepper *MATE* genes experienced purifying selection during evolution. In addition, collinearity analysis showed that *MATE* genes were highly conserved between pepper and potato. Analysis of *cis*-elements in *MATE* promoters and *MATE* expression patterns revealed that MATE proteins likely function in many stages of plant development, especially during fruit ripening, and when exposed to multiple stresses, consistent with the existence of functional differentiation between duplicated *MATE* genes. Together, our results lay the foundation for further characterization of pepper and potato *MATE* gene family members.

## 1. Introduction

The Multidrug and Toxic Compound Extrusion (MATE) protein family (also named Detoxification Efflux Carriers, DTXs) consists of essential multidrug transporters that can dispose and detoxify exogenous and endogenous toxins in development and response to various stresses [1]. Four other protein families function as multidrug transporters: the ATP-binding cassette (ABC) family, major facilitator superfamily (MFS), resistance-nodulation-division (RND) family, and small multidrug resistance (SMR) transporters [2]. MATE members play important roles as secondary drug resistance transporters, which extrude toxins using Na^+^ or H^+^ electrochemical gradients [3].

MATE proteins have been identified in bacteria, archaea, fungi, plants, and mammals; however, plant genomes encode far more MATE proteins than genomes from other kingdoms, possibly because of the wide range of metabolites that occur in plants [4]. A typical MATE protein is 440–550 amino acids in length and contains a conserved PF01554 domain, consisting of 12 alpha-helical transmembrane domains (TMs), as confirmed by an X-ray crystallography structure analysis of the NorM protein from the Gram-negative bacterium *Vibrio parahaemolyticus* [5,6] and the PROTEIN DETOXIFICATION 14 (DTX14) protein from *Arabidopsis* (*Arabidopsis thaliana*) [7].

To date, research about the function and regulation of plant *MATE* genes has been relatively limited. Several MATE transporters have been shown to transport diverse noxious compounds or secondary metabolites in a number of plant species, thereby regulating plant development and stress tolerance. Many MATE proteins have been confirmed to transport or accumulate secondary metabolites, including transport of alkaloids by tobacco (*Nicotiana tabacum*) jasmonate-inducible alkaloid transporter 1 (NtJAT1) and AtDTX1 [8,9,10]; anthocyanins and the epicatechin 3’-O-glucoside by *Arabidopsis* TRANSPARENT TESTA 12 (TT12)/DTX41 [11,12]; anthocyanins by grapevine (*Vitis vinifera*) anthoMATE1 (VvAM1) and VvAM3 [13,14]; proanthocyanidin precursors by barrelclover (*Medicago truncatula*) MtMATE1 [12]; nicotine or flavonoid by NtMATE1 [15], MtMATE2 [16]; AtDTX35/FLOWER FLAVONOID TRANSPORTER (FFT) [17]; salicylic acid (SA) by *Arabidopsis* ENHANCED DISEASE SUSCEPTIBILITY 5 (EDS5) [18,19,20,21]; abscisic acid (ABA) by AtDTX50/ABNORMAL SHOOT (ABS) 3-LIKE 2 (ABS3L2) [22]; hydroxycinnamic acid amides by AtDTX18 [23]. In addition, several MATE proteins are involved in aluminum detoxification or iron translocation, including *Arabidopsis* MATE [24,25], *Brassica oleracea* BoMATE [26], *Eucalyptus camaldulensis* EcMATE1 [27], *Arabidopsis* FERRIC REDUCTASE DEFECTIVE 3 (FRD3) [28,29], soybean (*Glycine max*) GmFRD3b [30], barley (*Hordeum vulgare*) HvAACT1 [31,32,33], rice (*Oryza sativa*) FRD3-LIKE 4 (FRDL4)/OsMATE4 [34,35], OsFRDL1/OsMATE10 [36,37,38], OsFRDL2/OsMATE40 [37], sorghum (*Sorghum bicolor*) SbMATE [39,40], rye (*Secale cereale*) ScFRDL1 and ScFRDL2 [41], wheat (*Triticum aestivum*) TaMATE1B [42], maize (*Zea mays*) ZmMATE1 [43,44], and *Arabidopsis* ZF14/AtABS4 [45,46,47].

With the advent of new sequencing technologies, the genomes of many organisms have been sequenced, which allowed the identification of many additional *MATE* genes. To date, a few *MATE* genes have been identified in species from other kingdoms; for instance, the human genome only has two *MATE* genes, *Solute carrier 47A1* (*SLC47A1*)*/MATE1* and *SLC47A2/MATE2-K* [48,49]. However, plant genomes contain large *MATE* gene families; for example, 45 or 55 have been identified in rice [50,51] and 49 in maize [52], which belong to the Poaceae family in the monocots. From the Leguminosae family in the eudicots, 70 were identified in *M. truncatula* [53], 117 in soybean [54], and 67 in pigeon pea (*Cajanus cajan*) [55]. In other eudicots, 71 were identified in poplar (*Populus trichocarpa*) [56], 73 in flax (*Linum usitassimum*) [57], 67 in Citrus sinensis [58], 56 in *Arabidopsis* [50], and 79–130 in four Brassicaceae species [59].

The Solanaceae family, also named nightshades, is one of the most economically and agronomically important plant families, including tomato (*Solanum lycopersicum*), tobacco, pepper (*Capsicum annuum*), potato (*Solanum tuberosum*) and other plants. Species in this family produce numerous secondary metabolites and toxic compounds, such as nicotine, capsaicin, hyoscyamine, and solanine; these compounds are used for foods or drugs [60]. The genomes of tomato [61], potato [62], and pepper [63,64] have been sequenced as high-quality assemblies and annotations and are frequently updated. An analysis of the tomato and potato genomes identified 67 *MATE* [4] and 48 *MATE* genes [65], respectively. However, little is known about the *MATE* family in pepper, and none have been experimentally characterized.

Here, our genome-level analysis of *MATE* gene families in the Solanaceae identified 42 *MATE* genes in pepper and 60 in potato. We classified plant *MATE* genes into five groups, based on their phylogenetic clustering, motif organization and exon–intron structures. In addition, we discovered that tandem duplications were the main drivers of gene family expansion in the pepper *CaMATE* family, and tandem duplications and segmental duplications drove expansion in the potato *StMATE* family, but both families were under purifying selection. Moreover, collinearity analysis showed that the pepper and potato *MATE* families were highly conserved. A detailed expression profile analysis revealed that pepper *CaMATE* genes exhibited functional diversity during development and opposite behavior in roots and leaves in response to different stresses. Our findings provide foundational information for the further validation of *MATE* genes in the Solanaceae.

## 2. Results

### 2.1. Identification of MATE Genes in the Pepper and Potato Genomes

To gain a comprehensive genome-wide overview of the *MATE* gene families in pepper and potato, we performed a Basic Local Alignment Search Tool for Protein (BLASTP) analysis using 56 *Arabidopsis* AtMATE proteins as queries [50], as well as an HMMER search for the Pfam MATE domain (PF01554). We focused our efforts on the pepper and potato genomes, as both plants are of great economic and agronomic value and have well-sequenced genomes with good annotation. We then filtered candidate genes according to the position of their MATE domain and the number of transmembrane domains (TMs), which we detected using the SMART, InterProscan and CDD databases. This analysis identified 42 putative *CaMATE* genes in pepper and 60 candidate *StMATE* genes in potato. According to their chromosomal locations, we designated these genes *CaMATE1*–*CaMATE42* and *StMATE1*–*StMATE60* (Supplementary Materials Table S1).

Table S1 lists the 42 *CaMATE* and 60 *StMATE* genes and their encoded proteins, including gene name and length, isoelectric point (pI) value, predicted molecular weight (MW), number of TMs and subcellular localization (Table S1). Pepper CaMATE proteins varied in length from 298 to 752 amino acids, contained 6 to 12 TMs, had a predicted MW from 32.4 to 82.5 kDa, and had pI values from 4.98 to 9.06. Likewise, potato StMATE proteins varied in length from 337 to 615 amino acids, contained 9 to 12 TMs, had a predicted MW from 37.1 to 65.9 kDa, and had pI values from 5.29 to 9.44. For comparison, the 56 *Arabidopsis* AtMATE proteins were 470 to 561 amino acids in length, contained 6 to 12 TMs, with predicted MW values ranging from 50.8 to 60.0 kDa, and pI values from 4.93 to 9.64. These results indicated that MATE proteins were more variable in size within the pepper and potato MATE family than in *Arabidopsis*. We predicted the subcellular location of each MATE protein using the WoLF PSORT database, which predicted that 36 out of the 42 pepper CaMATE proteins localize to the plasma membrane, with another three localizing to the vacuolar membrane, and three to the nucleus, a distribution that was similar to that of potato StMATE proteins (Table S1).

### 2.2. Phylogenetic Analysis and Structural Characterization of Pepper MATE Genes

**Figure 1:**
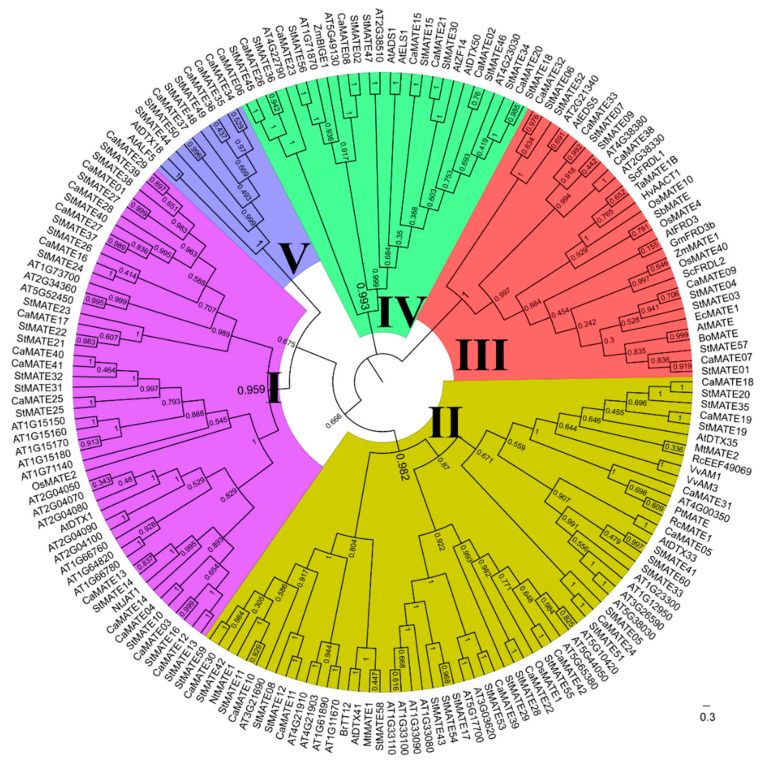
Phylogenetic relationship of 42 CaMATE, 60 StMATE, and 56 AtMATE proteins, along with another 25 functional published MATE proteins. The phylogenetic tree was constructed using MEGA X with the Maximum Likelihood method, and visualized with FigTree software. MATE proteins were classified into five distinct groups, as indicated by the different colors.

To explore the phylogenetic relationships and evolutionary history of the plant *MATE* gene family, we used MEGA X software to align the MATE domains from pepper, potato and *Arabidopsis* MATE family members along with another 25 functionally characterized plant MATE proteins with MUSCLE (Multiple Sequence Comparison by Log-Expectation), followed by phylogenetic analysis using the Maximum Likelihood method (Figure 1). We classified the 183 MATE proteins into five groups (Groups I–V), according to the topology of the phylogenetic tree, with high bootstrap values of 61.3, 88.3, 100, 98.3, and 98.9 (Figure 1), respectively, above the significance cutoff of 50. Intra-group bootstrap values were higher than between-group values (Figure 1). All five groups contained pepper, potato, and *Arabidopsis* MATE proteins, suggesting that the five groups formed before the divergence of the Brassicaceae and Solanaceae. The number of MATE proteins associated with each group was uneven in pepper and potato. Group I and Group II contained the largest number of MATE proteins, with 31 in pepper and 32 in potato.

**Figure 2:**
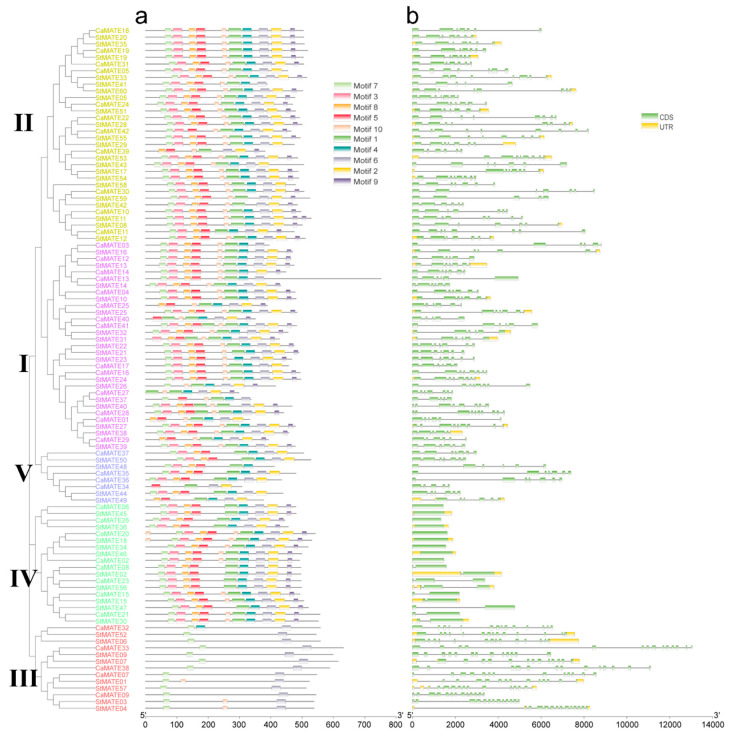
Conserved motifs in pepper and potato MATE proteins and their associated *MATE* gene structures. Left: phylogenetic tree of 42 CaMATE and 60 StMATE proteins replotted from Figure 1. (**a**) Conserved motifs of pepper and potato MATE proteins. Each colored box represents a protein motif identified by multiple EM for motif elicitation (MEME), and the box is placed at the appropriate position within the protein. MATE proteins are ordered according to the phylogenetic tree. (**b**) *MATE* gene structures in pepper and potato.

We analyzed MATE proteins for conserved motifs using the multiple EM for motif elicitation (MEME) suite, revealing ten conserved protein motifs whose organization within MATE proteins largely agreed with the phylogenetic tree (Figure 2a and Figure S1). In addition, motifs identified by MEME in pepper and potato MATE protein belonging to the same groups shared a high degree of conservation, as evidenced by their lower E-values, and similar motif numbers and organization. Groups I, II, IV and V shared similar protein domain composition and organizations that were distinct from those of Group III. For example, Group III members only had two motifs in common, but Group I, II, IV and V had 9–10 in common (Figure 2a). This observation suggests that Group III may have followed a different evolutionary trajectory compared to members of the other four groups.

To determine the extent of genomic structural diversity of *MATE* genes, we analyzed the exon–intron organization of the pepper and potato *MATE* genes, with the help of the Gene Structure Display Server (GSDS 2.0) website. The *MATE* gene family in both species showed a similar exon–intron structure within the same groups (Figure 2b), further validating the classification of *MATE* genes. Group I contained 48 *MATE* genes, with 44 (91.7%) having 5–7 introns; Group II contained 54 *MATE* genes, with 49 (91.7%) having 6–8 introns; Group III contained 19 *MATE* genes with 12–14 exons; Group V contained 10 *MATE* genes, with 4–6 introns (Figure 2b). Notably, the 27 *MATE* genes belonging to Group IV had 0–2 introns, suggesting a very different genomic structure for these genes (Figure 2b).

Our analysis demonstrated that pepper, potato and *Arabidopsis MATE* genes that belonged to the same group shared the same or a very similar arrangement of their functional motifs, intron patterns, and exon–intron structures, consistent with the phylogeny. Gene structures varied greatly among different groups, supporting the classification of the *MATE* family members.

### 2.3. Chromosomal Distribution and Duplication of Pepper and Potato MATE Genes

**Figure 3:**
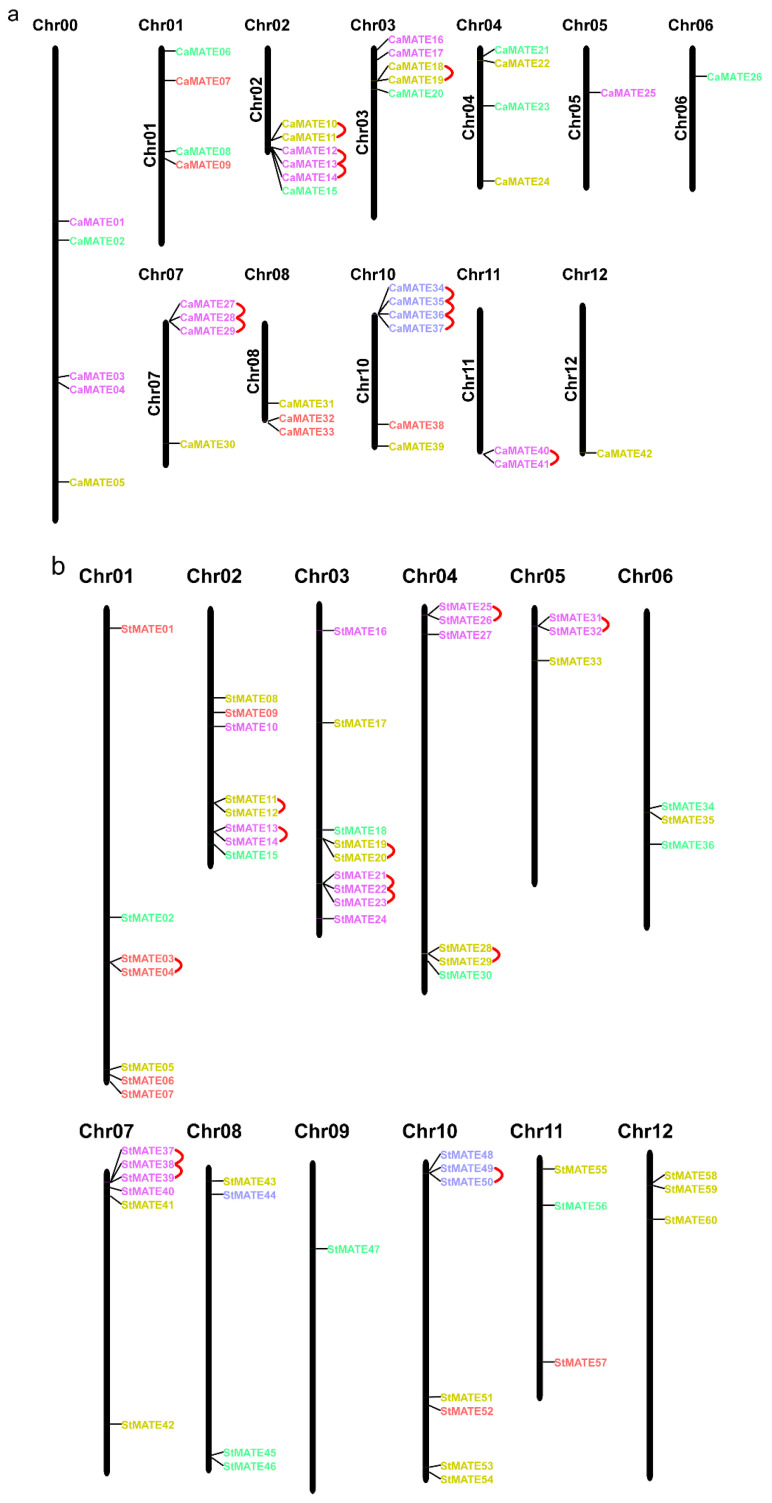
Chromosomal distribution of pepper and potato *MATE* genes. (**a**,**b**) pepper (**a**) and potato (**b**) locations of *MATE* genes on the chromosomes. The chromosome number is indicated above each chromosome (vertical bar). Chromosome lengths are based on information from the Ensembl database. Tandem-duplicated genes are connected by red arcs. *MATE* genes are color-coded according to the MATE group they belong to, in accordance with Figure 1.

To explore the relationship between pepper and potato *MATE* genes, we determined their chromosomal locations and whether they originated from gene duplication events. *MATE* loci were unevenly distributed in the pepper and potato genomes. We identified pepper *CaMATE* genes on all chromosomes, except chromosome 09. Several pepper chromosomes had four to six *CaMATE* genes (chromosomes 00, 01, 02, 03, 04, 07, and 10), while other chromosomes had one to three *CaMATE* genes (chromosomes 05, 06, 07, 11, and 12) (Figure 3a). In addition, we observed clusters of *CaMATE* genes on chromosomes 02, 07, and 10 (Figure 3a). Similarly, potato *StMATE* genes mapped to all 12 potato chromosomes, with six to nine *StMATE* genes on chromosomes 01, 02, 03, 04, 07, and 10 (Figure 3b).

Tandem-duplicated genes are defined as two paralogous genes that are separated by fewer than 10 intervening genes [50]. Using MCScanX, we identified 16 (38.1%) pepper *MATE* genes in five clusters that correspond to tandem duplications events (Figure 3a) and may have contributed to the expansion of the gene family. Among these tandem-duplicated genes, five pairs belonged to Group I, two pairs belonged to Group II, and three pairs belonged to Group V (Figure 3a). In potato, we identified 21 (35%) tandem-duplicated *StMATE* genes, comprising 12 gene pairs (Figure 3b). Of these, seven pairs belonged to Group I, with three, one, and one pairs that belonged to Groups II, III, and V, respectively (Figure 3b). These results suggested that tandem duplication played important roles in the expansion of the *MATE* gene family in both pepper and potato, but affected different *MATE* gene groups differently.

Interestingly, we failed to identify segmental duplication events among pepper *CaMATE* genes, in sharp contrast to potato, for which we identified 12 *StMATE* gene pairs resulting from segmental duplications (Figure 3b). Segmental-duplicated potato *StMATE* genes were observed in Groups I–IV, with six pairs in Group I, three pairs in Group II, one pair in Group III, and two pairs in Group IV. These results suggested that the *MATE* gene family has expanded by different mechanisms in pepper and potato. While tandem duplications contributed to *MATE* gene family expansion in both pepper and potato, segmental duplications only contributed to the expansion of the *MATE* family in potato, illustrating the similarities and differences between the two Solanaceous species.

**Figure 4:**
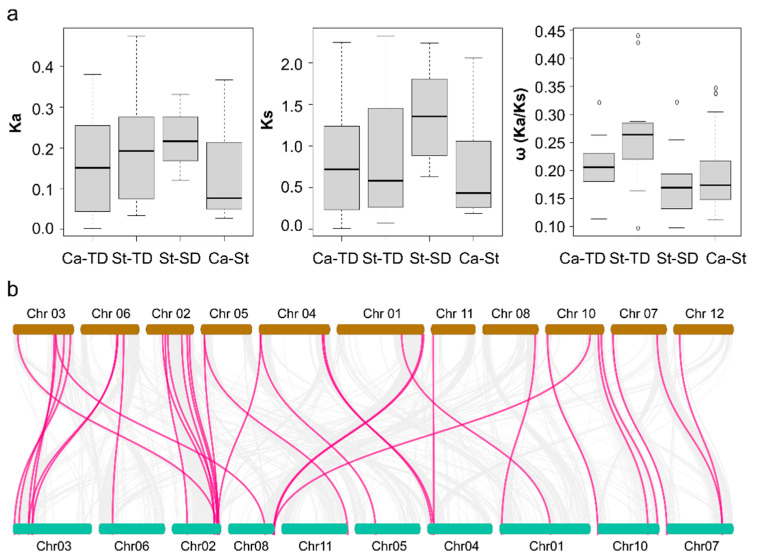
Collinearity analysis of *MATE* genes between pepper and potato. (**a**) Average value of Ka, Ks, and ω (Ka/Ks ratio). The x-axis indicates tandem duplication (TD) events in pepper (Ca) and potato (St), segmental duplication (SD) events in potato, and collinear pairs between pepper and potato (Ca-St). The y-axis shows Ka, Ks and Ka/Ks ratios of *MATE* genes for each pair. Boxplots were generated in R. (**b**) The potato chromosomes are in brown (top) and pepper chromosomes in green (bottom). Putative orthologous genes in their genomes are connected by lines and were identified using MCScanX. The innermost grey solid lines show collinear relationships between *MATE* genes. We identified 30 orthologous *MATE* gene pairs, indicated by magenta lines.

We then used the Ka (non-synonymous distance), Ks (synonymous distance), and ω, (Ka/Ks ratio) values to evaluate selective pressure exerted on the *MATE* family during evolution [66]. The neutral theory posits that ω values below 1 indicate purifying selection, while values around 1 represent neutral evolution and ω values above 1 indicate positive selection [67]. To explore the selective pressure imposed on tandem-duplicated *MATE* genes and their possible functional diversification in pepper and potato, we calculated the ω values for tandem- and segmental-duplicated *MATE* gene pairs. ω values were below 1 for all 34 *MATE* gene pairs, indicating that tandem and segmental-duplicated pepper and potato *MATE* genes experienced purifying selection (Figure 4a, Table S2).

We also calculated the mean ω value for tandem-duplicated *MATE* genes in pepper within each group: the ω value of Group II was 0.144, 0.228 for Group I and 0.227 for Group V, suggesting that *MATE* genes within Group II experienced much stronger purifying selection than those in Groups I and V. Likewise, we obtained similar ω values for potato tandem-duplicated pairs: the mean ω value was 0.144 for Group II pairs, 0.275 for Group I, 0.428 for Group III and 0.282 for Group V. However, potato segmental-duplicated *StMATE* gene pairs showed a mean ω value within Group I of 0.176, slightly lower than for tandem-duplicated pairs of the same group, suggesting that segmental-duplicated *StMATE* genes experienced much more relaxed selection than tandem-duplicated gene pairs in Group I. We saw no evidence of positive selection for any *MATE* gene pairs identified in either pepper or potato.

### 2.4. Collinearity Analysis of MATE Genes between Pepper and Potato

To assign orthologous gene pairs between pepper and potato, we performed a collinearity analysis using MCScanX [68] and TBtools software [69]. We identified 30 putative orthologous *MATE* gene pairs (Figure 4b). We detected 21 *CaMATE* genes on 10 of the 12 chromosomes of the pepper genome, which formed pairs with 27 potato *StMATE* genes mapping to 11 of the 12 chromosomes of the potato genome. Notably, pepper *CaMATE12* on chromosome 02 showed synteny with five collinear *StMATE* pairs in potato (Figure 4b). In terms of the five groups defined earlier, 12 putative orthologous pairs belonged to Group I and 10 pairs to Group II, with the remaining two, four, and one orthologous pairs belonging to Groups III, IV, and V, respectively (Figure 4b). We hypothesize that these putative orthologous pairs of *MATE* genes may share the same function in these two Solanaceous species.

### 2.5. Analysis of Cis-Regulatory Elements in MATE Promoters

**Figure 5:**
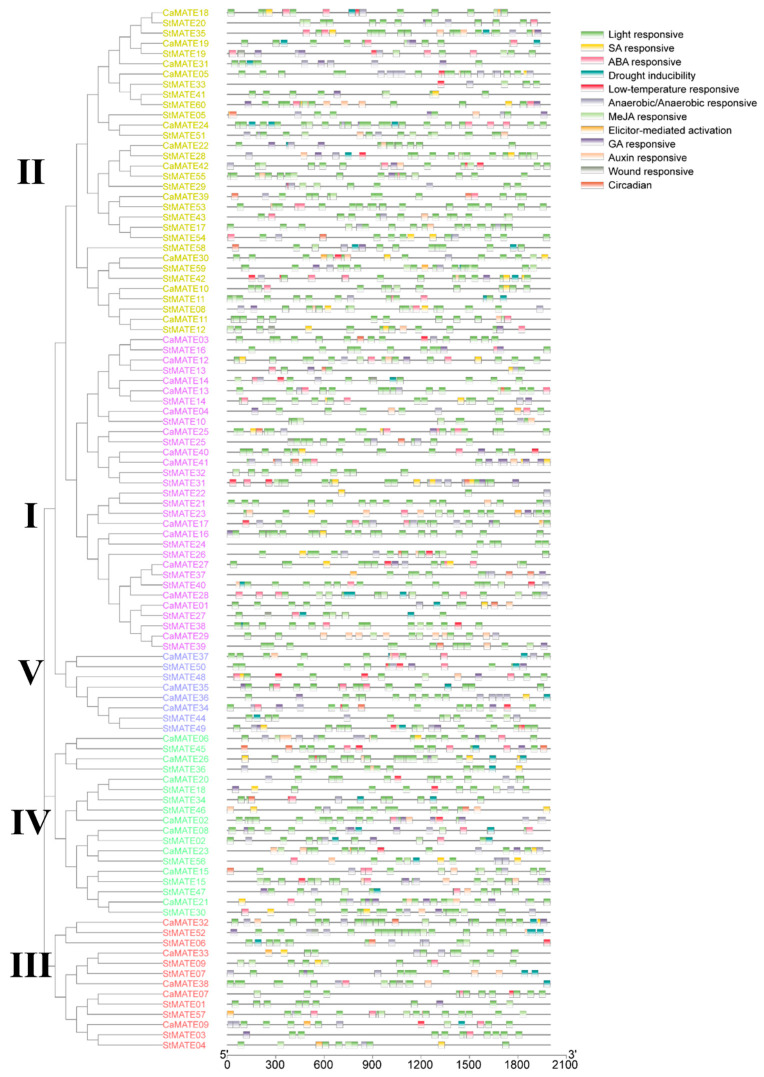
Predicted *cis*-regulatory elements in the promoters of pepper and potato *MATE* genes. The phylogenetic tree of the pepper CaMATE family is replotted from Figure 1. The *cis*-regulatory elements (CREs) in the 2 kb upstream regions of the 42 pepper *CaMATE* and 60 potato *StMATE* genes were predicted using the PlantCARE database. These CREs were divided into three types: development (including circadian-related and light-responsive elements), phytohormone (including ABA-responsive, auxin-responsive, GA-responsive, MeJA-responsive, and SA-responsive elements), and stress-responsive (including drought inducibility, low-temperature-responsive, elicitor-mediated activation, and wound-responsive elements).

*MATE* genes take part in plant development and defense responses [2,4,50]. To investigate their potential functions during plant development and upon exposure to various stresses, we performed an analysis of 2 kb of sequence upstream of each pepper and potato *MATE* gene. To this end, we used the PlantCARE website to predict the *cis*-regulatory elements (CREs) in this 2-kb region. This analysis identified 12 distinct CREs in the *MATE* promoters, including two development-related CREs, five phytohormone-responsive CREs, and five plant defense response-related CREs (Figure 5). The number of CREs was quite variable across the potato and pepper *MATE* genes, with the highest number seen in the pepper *CaMATE24* (39 CREs) and *CaMATE28* (36 CREs) promoters, and only three CREs in the potato *StMATE22* promoter (Figure 5). We identified 922 potential CREs in the pepper *CaMATE* promoters, including 523 elements related to development, 271 related to phytohormone responses, and 128 related to plant defense responses (Figure 5). The 523 development-related CREs consisted of 512 light-responsive and 11 circadian elements. The phytohormone-responsive CREs consisted of 91 abscisic acid (ABA)-responsive elements in 32 pepper *MATE* promoters, 34 auxin-responsive elements in 21 pepper promoters, 41 gibberellic acid (GA)-responsive elements in 24 pepper promoters, 80 methyl jasmonate (MeJA)-responsive elements in 27 pepper promoters, and 25 salicylic acid (SA)-responsive elements in 18 pepper promoters.

Of the 128 defense response-related CREs, we identified 24 drought-inducible sites (MYB-binding site or MBS) in 16 pepper promoters, 19 low-temperature-responsive elements (LTREs) in 16 pepper promoters (Figure 5), 7 AT-rich motifs (TAAAATACT) responsible for elicitor-mediated activation in seven pepper promoters, and 3 WUN-motifs (AAATTTCCT), responsible for wound-responsive expression, in the promoters of pepper *CaMATE2*, *CaMATE14* and *CaMATE38*. In addition, we identified 75 ARE and GC-motifs, mediating anaerobic or anoxic responses, in the promoters of 35 pepper *CaMATE* genes (Figure 5), suggesting that these genes may participate in responses to hypoxia.

The above results strongly suggested that pepper *CaMATE* genes participate in plant responses to multiple stresses. A similar analysis identified 1125 potential CREs in the promoters of the potato *StMATE* genes, including 641 related to development, 350 related to phytohormone responses, and 134 related to plant defense responses (Figure 5), underscoring the similar distribution of CREs between pepper and potato *MATE* genes. Many pepper and potato *MATE* promoters contained various CREs related to development, phytohormones, and plant defense (Figure 5), suggesting that these genes may play important roles during plant growth and response to environmental stresses.

### 2.6. Analysis of Pepper CaMATE Gene Expression Patterns

**Figure 6:**
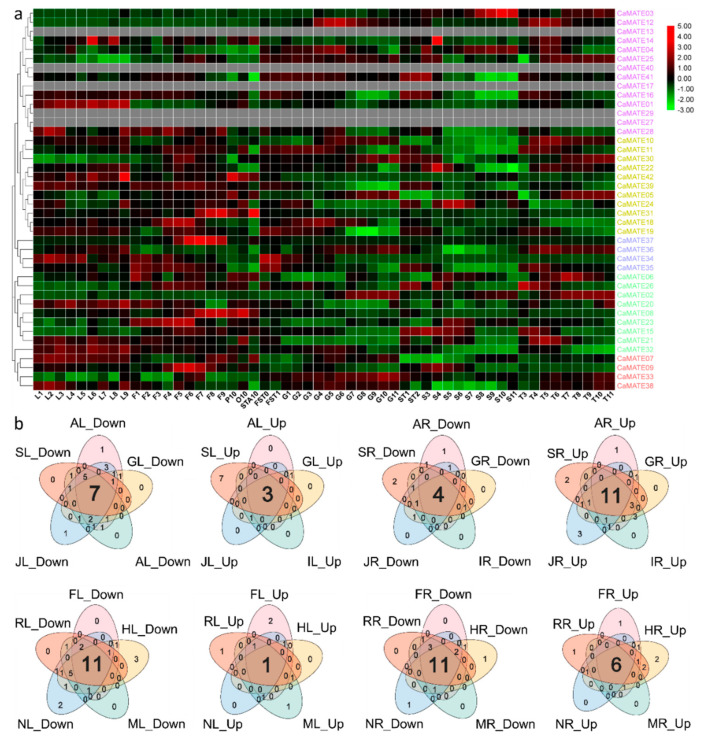
Expression of pepper *CaMATE* genes during plant development and in response to various stresses. (**a**) Tissue-specific expression data for pepper *CaMATE* genes were obtained from published data (http://www.hnivr.org/pepperhub). Red and green colors represent higher and lower expression, respectively. Pepper samples: L: leaf, F: flower, P: petal, O: ovary, STA: anther, FST: whole fruit, G: pericarp, T: placenta, ST: placenta, and seed, S: seed. Heat map of the expression data for *MATE* genes in the selected 54 pepper tissues. The heat map with phylogenetic tree was drawn with R. (**b**) Venn diagrams of *CaMATE* expression in response to phytohormones and stress. A: ABA-treated; S: SA-treated; J: JA-treated; I: IAA-treated; G: GA-treated; F: freezing-treated; R: H_2_O_2_-treated; N: NaCl-treated; M: mannitol-treated; H: heat-treated; Up: up-regulated genes; Down: down-regulated genes. L: leaves; R: roots. The two-letter code lists the treatment first, then the tissue. The numbers given in the Venn diagram represent the numbers of up-/down-regulated genes.

To assess the role of pepper and potato *MATE* genes in plant development, we turned to transcriptome deep-sequencing (RNA-seq) datasets from the PepperHub database [70], which we collected and analyzed as previously described [71]. We compiled the expression profiles of all pepper *CaMATE* genes across 54 different tissues and organs and ordered the genes according to their positions within the phylogenetic tree. We then visualized transcript levels as a heatmap, which illustrated the differences in expression patterns observed for *CaMATE* genes (Figure 6a). When applying a selection criterion of fifty Reads Per Kilobase of transcript, per Million mapped reads (RPKM) in at least one tissue, we identified 16 highly expressed *CaMATE* genes from all five phylogenetic groups. Of note was the observation that highly expressed genes exhibited different expression patterns in different pepper tissues (Figure 6a). Within the same group, *CaMATE* genes showed distinct expression profiles in different tissues (Figure 6a), suggesting subfunctionalization or functional diversification.

Our analysis also highlighted a set of five genes (*CaMATE13*/*17*/*27/29/40*), from Group I with expression levels below 1 RPKM in all samples, suggesting that these may be pseudogenes. Interestingly, four of these low-expressed genes (*CaMATE13/27/29/40*), were derived from tandem duplication, indicating that they may be undergoing pseudogenization. We identified 37 *CaMATE* genes with preferential expression in a single pepper tissue such as leaf, flower, pericarp, placenta, or seed (Figure 6a), hinting at their involvement in growth and development of the corresponding tissues. We detected seven highly expressed *CaMATE* genes in leaves, eleven in flowers, four in pericarp, eight in placenta and seven in seeds (Figure 6a), suggesting a function as tissue- or organ-specific regulators. Further investigation indicated that tandem-duplicated *CaMATE* genes were differentially expressed in the selected samples (Figure 6a), suggesting their functional differentiation.

### 2.7. Expression Analysis of Phytohormone- and Stress-Responsive Pepper CaMATE Genes

*MATE* genes take part in plant response to abiotic and biotic stresses [2]. To explore whether *CaMATE* genes are involved in plant responses to environmental stresses, we analyzed the expression pattern of *CaMATE* genes in pepper roots and shoots exposed to five phytohormones (ABA, GA, indole-3-acetic acid (IAA), JA, and SA) and five stress conditions (freezing, H_2_O_2_, salt, mannitol and heat stress), obtained from the PepperHub database [70,72]. Our analysis showed that 35 *CaMATE* genes were regulated by phytohormones and the stresses tested here (Figure 6b). The majority of *CaMATE* genes were regulated by more than one phytohormone or stress treatment, with only *CaMATE16* being induced by JA in roots, and *CaMATE05* being repressed by JA in shoots (Figure 6b). We identified 25–28 *MATE* genes that were regulated by phytohormones in roots, compared to 20–28 *MATE* genes in leaves (Figure 6b). In roots, 10–21 genes were down-regulated, and 7–10 genes were up-regulated compared with untreated controls. We saw the opposite pattern in shoots, where more *MATE* genes were repressed than induced in response to phytohormone treatments (Figure 6b).

In roots, we observed the induction of *CaMATE* genes that belonged to each of the five phylogenetic groups: five genes from Group I (*CaMATE01/03/04/14/17*), three genes from Group IV (*CaMATE06*/*20*/*26*), two genes from Group II (*CaMATE19* and *CaMATE24*) and one gene from Group III (*CaMATE09*) in response to all phytohormones. In addition, two Group III (*CaMATE07/32*), one Group I (*CaMATE41*), and one Group II (*CaMATE18*) gene were repressed by all phytohormones in roots (Figure 6b). In leaves, only two Group I *MATE* genes (*CaMATE01/28*) and one Group II gene (*CaMATE18*) were induced by treatment with all phytohormones. Finally, three Group I (*CaMATE03/17/14*), two Group II (*CaMATE19/24*), two Group IV (*CaMATE23/26*) genes, and one Group III (*CaMATE33*) gene were repressed by treatment with the phytohormones in leaves (Figure 6b). These results showed that pepper *CaMATE* genes are differentially expressed in response to phytohormones and in a tissue-specific manner.

We also determined the gene expression profile of pepper *CaMATE* genes in response to stress such as freezing (F), H_2_O_2_ (R), salt (NaCl, N), mannitol (M), and heat (H), leading to the identification of 35 *CaMATE* genes that were differentially expressed in response to these treatments. As with the phytohormones above, the majority of *CaMATE* genes were regulated by more than one treatment, with only *CaMATE32* and *CaMATE38* being repressed by heat and salt stress in shoots, and *CaMATE36* being repressed by freezing in roots (Figure 6b). There were 23–30 *CaMATE* genes that were regulated by various stresses in roots, compared to 22–29 *MATE* genes regulated in leaves (Figure 6b). In roots, 16–19 genes were down-regulated, while 7–10 *CaMATE* genes were up-regulated in response to stress treatments. Again, this pattern was opposite in shoots, with more *MATE* genes being repressed (16–25 *MATE* genes) than induced (3–6 *MATE* genes) (Figure 6b). In roots, five Group I genes (*CaMATE01/03/04/12/14*), three Group IV genes (*CaMATE06/09/20*), two Group II genes (*CaMATE19/24*) and one Group III (*CaMATE09*) gene were induced in all stress conditions. Likewise, three Group II genes (*CaMATE05/18/39*), two Group III genes (*CaMATE07/32*), and one Group I (*CaMATE41*) gene were repressed by all stresses in roots (Figure 6b). In leaves, only the group I gene *CaMATE28* was induced by all stresses. In addition, four Group IV genes (*CaMATE06/21/23/26*), three Group I genes (*CaMATE03/12/14*), three Group II genes (*CaMATE19/22/24*), and one Group III (*CaMATE09*) gene were repressed by all stresses in leaves (Figure 6b). The above results showed that *CaMATE* genes may be involved in plant responses to various stresses and may have opposite regulatory roles in roots and leaves. Indeed, *CaMATE* genes may take part in pepper responses to freezing, H_2_O_2_, salt, mannitol, and heat stresses. Collectively, the similarity in expression patterns of *CaMATE* genes in response to phytohormones and stresses (Figure 6b) suggests a shared response brought upon by phytohormones and stresses.

**Figure 7:**
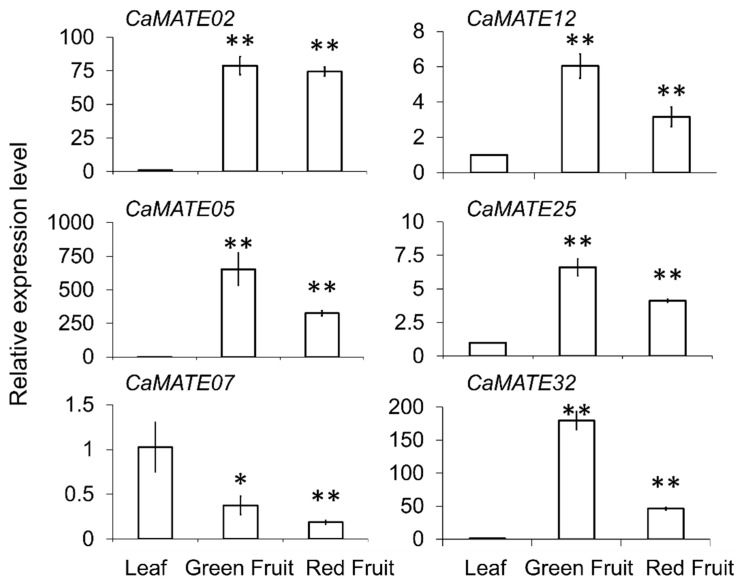
Expression of *CaMATE* genes during development. Quantitative reverse transcription PCR (qRT-PCR) analysis of six *CaMATE* genes in leaf and fruit tissues. *CaMATE* transcript levels were normalized using *CaUBI-3* as the internal reference. Each data point represents the average of three biological repeats. * *p* < 0.05; ** *p* < 0.01 by Student’s *t*-test.

To elucidate *CaMATE* functions during pepper fruit ripening, we selected several *CaMATE* genes for RT-qPCR analysis, which demonstrated that *CaMATE02/05/12/25*/30 showed preferential expression in green and red fruit tissues (Figure 7), consistent with the data from the PepperHub RNA-seq datasets (Figure 6). These observations suggested that *CaMATE* genes participate in plant fruit development, when pepper produces many metabolites during fruit ripening.

## 3. Discussion

MATE proteins are ubiquitous in nearly all kingdoms. In plants, they participate in diverse functions that regulate plant development and adaptation to stresses, by transporting and sequestering harmful substances and secondary metabolites. Although *MATE* genes have been identified in several plant genomes [50,51,52,53,54,55,56,57,58,59], little is known about the function of MATE transporters in Solanaceous species, which are of high economic and agronomic importance. Here, we performed a comprehensive genome-wide identification of *MATE* genes in pepper and potato, two members of the Solanaceae, identifying 42 *CaMATE* and 60 *StMATE* genes. Subsequently, we combined phylogenetic analysis, gene structure characterization, and expression pattern analysis to elucidate the evolution of *MATE* genes and their potential functions, which will contribute to our understanding of MATE transporter functions in the Solanaceae.

### 3.1. MATE Gene Family Conservation in the Solanaceae

Our analysis revealed 42 *CaMATE* genes in pepper and 60 *StMATE* genes in potato (Table S1). The *StMATE* gene family has previously been reported to consist of 48 members [65], but we attribute the higher numbers identified here to the recent update of the potato genome annotation. The number of *MATE* genes in pepper and potato was comparable to that in tomato, which has 60 *MATE* genes [4]. The *MATE* gene family has greatly expanded in plants relative to other kingdoms [48,49], suggesting their diverse and vital roles in plants.

Pepper CaMATE proteins varied from 298 to 752 amino acids, while potato StMATE proteins consisted of 337 to 615 amino acids, much longer than the range seen in *Arabidopsis*, with AtMATE proteins ranging from 470 to 561 amino acids [50], suggesting a higher diversity and complexity in the Solanaceae. Our analysis predicted that most MATE proteins localize to the plasma membrane, which would be consistent with their roles as transporters of toxic compounds [7], thereby conferring resistance to the toxin.

A phylogenetic tree constructed using 42 CaMATE, 60 StMATE, 56 AtMATE, and another 25 functionally characterized MATE proteins from other plant species classified MATE family members into five groups (Figure 1), which we validated based on their gene structures and the organization of their encoded functional motifs (Figure 2). In agreement with the phylogenetic analysis, gene structures, the number of exons, the number of TM domains, and the predicted subcellular locations showed higher similarity within each group than between groups, supporting our classification of MATE members. MATE family members from Group III only displayed two conserved motifs, but had the most exons relative to all other groups (Figure 2), indicating large structural differences in the *MATE* genes and variation in the function of the encoded proteins.

### 3.2. Tandem Duplications Contributed to MATE Gene Expansion between Pepper and Potato

The pepper and potato *MATE* genes were unevenly distributed across the chromosomes of their respective genomes, as already observed with the tomato *SlMATE* family [4], indicating a possible aneuploidy event, in addition to the whole genome triplication event that occurred in these Solanaceous species [61,64,73].

Intra- and inter-synteny and collinearity analysis suggested that pepper *CaMATE* genes may have expanded by tandem duplication, as in tomato [4], resulting in the tight linkage of *MATE* genes in clusters in the Solanaceae (Figure 3 and Figure S1), and implying that tandem duplications may have contributed to the expansion of the *MATE* gene family in the Solanaceae. The ω values for tandem-duplicated pepper *CaMATE* genes ranged from 0.113 to 0.321, comparable to the 0.097 to 0.440 range seen for potato *StMATE* genes (Figure 4, Table S2). Importantly, these values were all less than 1, indicating purifying selection during evolution of the *MATE* gene family in pepper and potato. Notably, we detected no obvious segmental duplications among pepper *CaMATE* genes (Figure 1), but identified 12 such segmental duplication pairs of *StMATE* genes in potato (Figure 3b) and tomato [4], suggesting that expansion of *MATE* gene families may be driven by distinct mechanisms among Solanaceous species. In addition, there was a non-uniform number of tandem-duplicated *MATE* genes within groups (Figure 3 and Figure S1). Indeed, we detected tandem duplication in Groups I, II and V *MATE* genes in pepper (Figure 4, Table S2), and in Groups I, II, III and V for potato, while potato segmentally duplicated *MATE* genes belonged to Groups I, II, III and IV (Figure 4, Table S2). These results suggest that different groups have undergone diversification in pepper and potato, especially genes belonging to groups III and IV.

We classified pepper and potato *MATE* paralogous genes, which were derived from tandem and segmental duplications and showed similar motif organization and exon–intron structures, into the same group, with strong support from phylogenic bootstrapping values (Figure 1 and Figure 2). This suggested that they experienced purifying selection, without any domain gain or loss. After duplication, the duplicated genes will undergo possible subfunctionalization, neo-functionalization, or non-functionalization [74]. In Group I, we identified four *CaMATE* genes (*CaMATE13/27/29/40*) originating from tandem-duplicated pairs with *CaMATE12/14/28/41* being expressed below 1 RPKM across all tissues tested (Figure 6), making them good candidates for pseudogenes that experienced non-functionalization after duplication. In Group II, the two duplicated *CaMATE* gene pairs (*CaMATE10*/*11*, *CaMATE18*/*19*) were expressed at their highest levels in flowers and the placenta, respectively, but at different developmental stages (Figure 6), suggesting subfunctionalization after duplication. In Group V, a cluster of duplicated *CaMATE* genes *CaMATE34/35/36/37* were preferentially expressed in flowers or the placenta (Figure 6), suggesting their subfunctionalization during evolution. Overall, *MATE* gene duplications drove diversification of gene expression; this may have affected plant development and helped plants adapt to changes in the environment in the Solanaceous species studied here.

### 3.3. MATE Function and Gene Expression

Changes in gene expression are routinely used to assess gene function during development and after exposure to stress conditions [75]. Accordingly, we used the preferential expression of pepper *CaMATE* genes in various tissues and stressed samples to predict their functional roles [71]. MATE proteins have been reported to extrude primary and secondary metabolites, such as organic molecules, terpenoids, alkaloids, phenols, and phytohormones [2]. In addition, several plant *MATE* genes have been shown to take part in plant development and response to stresses, by means of excreting toxic compounds. An analysis of RNA-seq data across 54 tissues or organs from PepperHub [70] established that many *CaMATE* genes were preferentially expressed in reproductive tissues, including flowers, pericarp, placenta, and seeds (Figure 6), suggesting that these genes may be involved in reproductive development. The Group II *CaMATE* genes, *CaMATE18* and *CaMATE39*, showed high expression in flowers, while *CaMATE30* expression was high during the G11 stage of pericarp development (Figure 6), indicating that these CaMATEs may have specific and narrow functions in reproductive tissues. Finally, *CaMATE37* from Group V reached a peak in expression at the F7 stage in flowers, with almost no expression in other tissues or organs (Figure 6), suggesting that CaMATE37 may participate in the specification of floral organs, especially at the F7 stage.

We also screened the promoters of all pepper *CaMATE* genes for *cis*-regulatory elements, resulting in the identification of 922 CREs, with 523 CREs related to development, and 399 CREs related to phytohormones or stress (Figure 5), suggesting that *CaMATE* genes may play important roles in plant development and adaptation to environmental conditions. We observed strong connections between plant responses to phytohormones and stress [76,77]. Our results showed that phytohormone- and stress-related CREs were abundant across *CaMATE* promoters, suggesting that *CaMATE* genes contribute to plant responses to environmental stresses. A more detailed analysis of gene expression profiles across the 54 developmental stages and 24 treatments available at PepperHub will lay a solid foundation for the functional characterization of pepper *CaMATE* genes. Overall, *CaMATE* genes exhibited various and highly diversified expression profiles, especially in reproductive tissues (Figure 7), implying that *CaMATE* genes may have significant and complex functions in pepper development and in response to environmental stimuli.

Plants are continuously exposed to many environmental stresses; they have therefore evolved multiple stress response mechanisms to deal with a changing environment [78]. The excretion of metabolites and toxic compounds by transporters is one such regulatory mechanism that leads to improved plant stress resistance. Phylogenetic relationships between the pepper and potato *MATE* gene family may be used to predict putative gene functions, according to published functional characterization of plant *MATE* genes [4]. To date, the function of only a few *MATE* genes has been experimentally validated.

Based on phylogeny, Group I contained 14 *CaMATEs*, 17 *StMATEs*, 17 *AtMATEs,* tobacco (*Nicotiana tabacum*) *NtJAT1s*, and rice (*Oryza sativa*) *OsMATE2* (Figure 1), which was the biggest *MATE* gene subfamily in plants. To date, NtJAT1 and AtDTX1 have been confirmed to transport alkaloids from the cytosol to the vacuole, regulating plant development and disease resistance [8,9,10], suggesting that Group I *MATE* genes may participate in the transport of alkaloids and plant response to disease. Group II contained 11 *CaMATEs*, 21 *StMATEs*, 22 *AtMATEs*, and another 10 *MATE* genes (Figure 1). Several Group II *MATE* genes have been reported to transport secondary metabolites, such as proanthocyanin, flavonoids, and nicotine. *Arabidopsis* TT12/DTX41 mediates anthocyanin [11] and epicatechin 3′-O-glucoside [12] transport, and was shown to control the sequestration of flavonoids in the vacuole, thereby affecting seed coat pigmentation [79]. BrTT12 from rapeseed (*Brassica rapa*) also plays a role in seed coat pigmentation [80]. MtMATE1 localizes to the tonoplast and transports proanthocyanidin precursors to the seed coat [12]. NtMATE1 and MtMATE2 transport nicotine or flavonoids from the cytosol to the vacuole [15,16]. Grapevine (*Vitis vinifera*) VvAM1 and VvAM3 appear to transport anthocyanins [13,14]. *Arabidopsis* DTX35/FFT was experimentally confirmed to be a flavonoid transporter [17], while DTX33 serves as a chloride channel that plays important roles in turgor regulation during stomatal movement [81]. Although the pepper and potato MATEs have not been functionally characterized, their similarity to MATEs from other species suggests that Group II MATEs may mediate the transport and accumulation of secondary metabolites.

Group III contained 5 *CaMATEs*, 8 *StMATEs*, and 6 *AtMATEs*. The few functionally characterized *MATE* genes from this group have been suggested to be involved in aluminum detoxification or iron translocation [2], and included *AtMATE* [24,25] and *AtFRD3* [28], *BoMATE* [26], *EcMATE1* [27], *GmFRD3b* [30], *HvAACT1* [31,32,33], *OsFRDL1*/*OsMATE10* [36,37,38], *OsFRDL4*/*OsMATE4* [34,35], and *OsFRDL2*/*OsMATE40* [37], *SbMATE* [27,39,40], *ScFRDL1* and *ScFRDL2* [41], *TaMATE1B* [42], and *ZmMATE1* [43,44]. The results above suggested that Group III *MATE* genes may constitute the best candidates for aluminum detoxification or iron translocation. By contrast, Group IV contained 8 *CaMATEs*, 10 *StMATEs*, and 9 *AtMATEs*. *Arabidopsis BUSH-AND-CHLOROTIC-DWARF 1* (*BCD1*)*/ZARIZ* (*ZRZ*)*/ABS4* is involved in organ initiation, iron homeostasis, and hypocotyl cell elongation [45,46,47], while *ACTIVATED DISEASE SUSCEPTIBILITY 1* (*ADS1*)*/ABS3/ ALTERED DEVELOPMENT PROGRAM 1* (*ADP1*) was reported to negatively regulate plant disease resistance and hypocotyl cell elongation [47,82,83,84,85]. *Arabidopsis EARLY LEAF SENESCENCE 1* (*ELS1*)/*ABS3L1* and *DTX50/ABS3L2* were shown to regulate cell elongation [22,86]. Group V contained 4 *CaMATEs*, 4 *StMATEs*, and 2 *AtMATEs* genes. *Arabidopsis* DTX18 functions in the export of hydroxycinnamic acid amides to the leaf surface, inhibiting the germination of *Phytophthora infestans* spores [23], while ABERRANT LATERAL ROOT FORMATION 5 (ALF5) was reported to be an efflux transporter for the protection of roots from toxic compounds [87].

The above results provide valuable information for further functional characterization of *MATE* genes during development and under stress conditions.

## 4. Materials and Methods

### 4.1. MATE Gene Identification

To identify *MATE* gene family members in pepper (*Capsicum annuum*) and potato (*Solanum tuberosum*), we used the 56 *Arabidopsis* AtMATE proteins [50] as queries for Basic Local Alignment Search Tool for Protein (BLASTP) against the pepper and potato proteomes [62,63,64]. In addition, we downloaded the Pfam entry PF01554 for the MATE domain from the Pfam database [88] and used it to search for MATE candidates in the pepper and potato proteomes from the Ensembl database [89], using HMMER 3.0 software [90], with an E-value cutoff of 10^−5^, as previously described [71,91]. Candidate MATE proteins were further examined for the presence of the complete MATE domain, followed by protein scans through the SMART [92], CDD [93], InterProscan [94], and Pfam [88] databases.

### 4.2. Gene Structure and Domain Combinations Analysis

We predicted the *MATE* gene exon–intron organization through the Gene Structure Display Server (GSDS 2.0) (http://gsds.cbi.pku.edu.cn) [95], and TBtools [69]. MATE protein sequences were also used to search the SMART [92], InterProscan [94], and MEME [96] databases to detect functional domains.

### 4.3. Gene Promoter Cis-Regulatory Elements and Protein Subcellular Predictions

The *MATE* gene promoters were retrieved as the 2-kb sequence upstream of the coding regions, and were scanned through the PlantCARE database (http://bioinformatics.psb.ugent.be/webtools/plantcare/html) [97] to predict putative *cis*-regulatory elements. We determined the predicted subcellular localization of MATE proteins using the WoLF PSORT database (https://psort.hgc.jp).

### 4.4. Phylogenetic Analysis

We aligned the protein sequence of all MATE domains using MUSCLE, followed by phylogenetic tree construction using the maximum likelihood method in MEGA X [98], with the most suitable substitution pattern (LG + G + F), estimated by 56 different amino acid substitution models (Table S3), pairwise deletions and 1000 bootstraps. The phylogenetic tree was visualized using FigTree software.

### 4.5. Gene Duplication Synteny Analysis

The determination of intra-genomic syntenic and inter-genomic collinearity blocks in the Solanaceae was performed by employing MCScanX software [68] with default parameters. Tandem and segmental duplications were detected as previously described [50,71]. Tbtools software was used to visualize and illustrate the results [69].

### 4.6. Gene Expression Analysis

The tissue- and stress-specific expression patterns of pepper *MATE* genes were obtained from previously published transcriptome deep sequencing (RNA-seq) datasets (http://pepperhub.hzau.edu.cn) [70]. We identified differentially expressed genes and clustered the results using R software as previously described [99,100,101].

### 4.7. Quantitative RT-PCR Analysis

We harvested leaf and fruit tissue from the pepper cultivar Capsicum 6421 and stored *the tissue* at −80 °C [102,103] until RNA extraction. We performed quantitative reverse transcription PCR (qRT-PCR) analysis as previously described [102,104,105]. Primers used for pepper MATE gene expression analysis were obtained from the qprimerDB database [106] (Table S4), with CaUBI-3 as the internal control.

## 5. Conclusions

This comprehensive analysis of the *MATE* gene family in two economically and agronomically important species from the Solanaceae identified 42 *CaMATE* genes in pepper and 60 *StMATE* genes in potato, with an uneven distribution across chromosomes. Our phylogenetic analysis and gene structure analysis grouped these plant *MATE* genes into five groups, which showed notable conservation within each group, validating our classification scheme. Intra-genome synteny analysis indicated that tandem duplications played an important role in shaping the evolution of the pepper *CaMATE* gene family, while inter-genome collinearity analysis revealed the putative orthologs between the two Solanaceous species. Estimation of ω values demonstrated that *MATE* genes were under purifying selection. The analysis of *cis*-elements in the *MATE* promoters and *MATE* gene expression patterns highlighted their functional diversification in plant reproductive development and adaptation to diverse environmental stimuli, with functional differentiation between tandem-duplicated genes. In particular, examination of their expression patterns suggested that *CaMATE* genes might participate in plant fruit development, as pepper produces many metabolites during fruit ripening. This study provides a comprehensive and systematic characterization of the pepper and potato *MATE* gene families and will assist the continuing investigation of MATE functions in the Solanaceae.

## Supplementary Materials

Supplementary materials can be found at https://www.mdpi.com/2223-7747/9/11/1448/s1. Figure S1: Motif sequences of the pepper and potato MATE proteins. MATE protein motif sequences were identified using MEME database. E-value indicates motif statistical significance. Sites indicated the amount of sites contribution in each motif. Width indicates the amount of amino acid in each motif. Table S1: Characteristic features of 42 CaMATE and 60 StMATE genes. Table S2: Ka, Ks and Ka/Ks ratios of segmental duplication pairs between pepper and potato. Table S3: Amino acid substitution models estimation of the maximum likelihood. Table S4: List of CaMATE genes primers used for qRT-PCR.

## Abbreviations

ABA	abscisic acid
Ca	*Capsicum annuum*
CRE	cis-regulatory element
GA	gibberellin
GSDS	Gene Structure Display Server
HMM	hidden Markov model
IAA	indole-3-acetic acid
Ka	non-synonymous distance
Ks	synonymous distance
MATE	Multidrug and Toxic Compound Extrusion
MeJA	methyl jasmonate acid
MEME	multiple EM for motif elicitation
MUSCLE	Multiple Sequence Comparison by Log-Expectation
MW	molecular weight
Os	Oryza sativa
pI	isoelectric point
qRT-PCR	quantitative reverse transcription PCR
SA	salicylic acid
St	*Solanum tuberosum*
