# Supplementary material for: Identification and Expression of the Multidrug and Toxic Compound Extrusion (MATE) Gene Family in Capsicum annuum and Solanum tuberosum
Source: Plants (Basel). 2020 Oct 27;9(11):1448. doi: 10.3390/plants9111448 (PMC7716203; doi:10.3390/plants9111448)
Supplement: Supplementary file 1 [file plants-09-01448-s001.zip › plants-975718-supple proofreading/plants-975718-supplementary proofreading.docx]

Supplemental Data

**Identification and Expression of the *Multidrug and Toxic Compound Extrusion* (*MATE*) Gene Family in *Capsicum annuum* and *Solanum tuberosum***


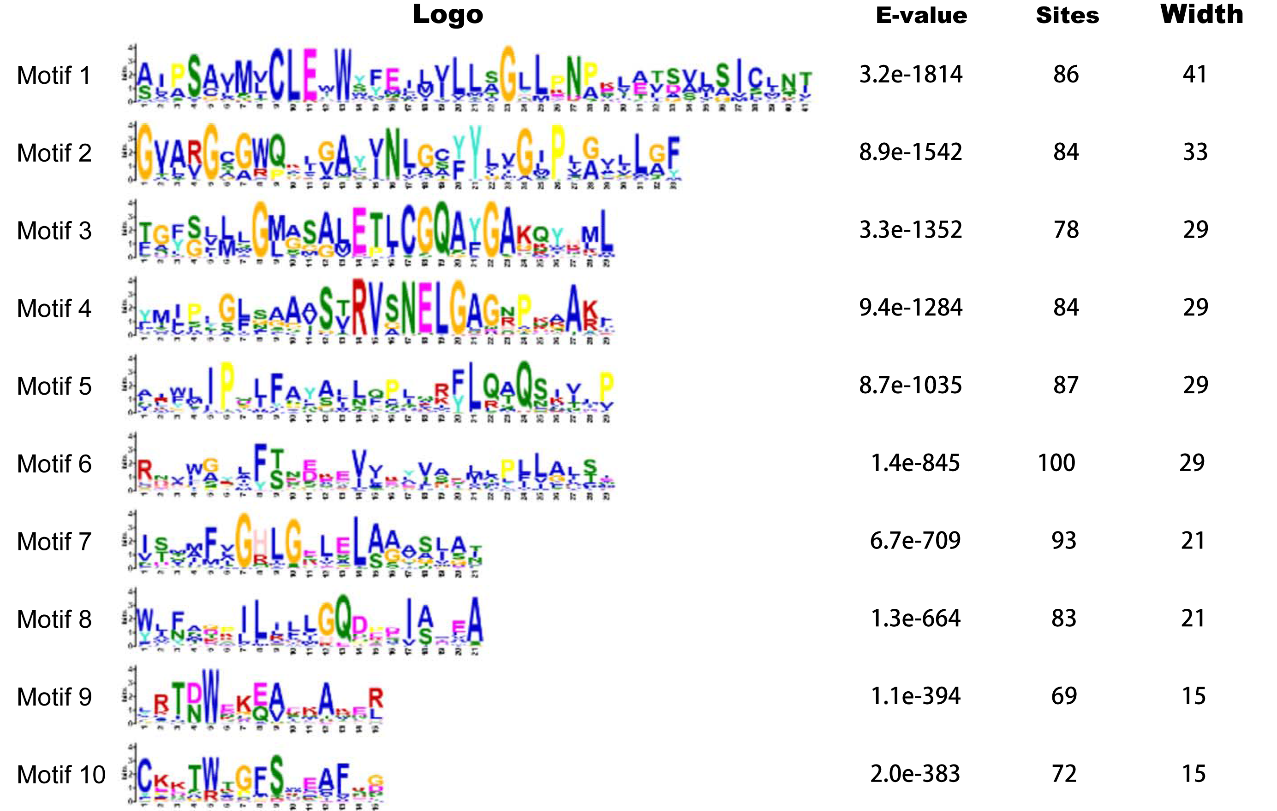


**Figure S1. Motif sequences of the pepper and potato MATE proteins.** MATE protein motif sequences were identified using MEME database. E-value indicates motif statistical significance. Sites indicated the amount of sites contribution in each motif. Width indicates the amount of amino acid in each motif.

**Table S1. Characteristic features of 42 *CaMATE* and 60 *StMATE* genes.**

**Table S2. Ka, Ks and Ka/Ks ratios of segmental duplication pairs between pepper and potato**

**Table S3. Amino acid substitution models estimation of the Maximum Likelihood.**

**Table S4. List of *CaMATE* genes primers used for qRT-PCR.**
